# Recent trends in rapid diagnostic techniques for dermatophytosis

**DOI:** 10.1080/23144599.2020.1850204

**Published:** 2020-12-17

**Authors:** Hassan Aboul-Ella, Rafik Hamed, Heidy Abo-Elyazeed

**Affiliations:** a Department of Microbiology, Faculty of Veterinary Medicine, Cairo University, Giza Egypt; b Bacteriology Biotechnology Diagnostics Department, Institute for Evaluation of Veterinary Biologics (CLEVB), Agricultural Research Center (ARC), Cairo, Egypt

**Keywords:** Dermatophytes, rapid diagnosis, lateral flow, immune-chromatographic kit, molecular mycology

## Abstract

Dermatophytosis is a common contagious disease of both humans and animals. It is caused by a group of filamentous fungi known as dermatophytes, including several genera and various species. An accurate diagnosis of dermatophytes as a causative agent of a skin lesion requires up to one month of conventional laboratory diagnostics. The conventional gold standard diagnostic method is a direct microscopic examination followed by 3 to 4 weeks of Sabouraud’s dextrose agar (SDA) culturing, and it may require further post-culturing identification through biochemical tests or microculture technique application. The laborious, exhaustive, and time-consuming gold standard method was a real challenge facing all dermatologists to achieve a rapid, accurate dermatophytosis diagnosis. Various studies developed more rapid, accurate, reliable, sensitive, and specific diagnostic tools. All developed techniques showed more rapidity than the classical method but variable specificities and sensitivities. An extensive bibliography is included and discussed through this review, showing recent variable dermatophytes diagnostic categories with an illustration of weaknesses, strengths, and prospects.

## 1. Introduction

**Figure 1.**
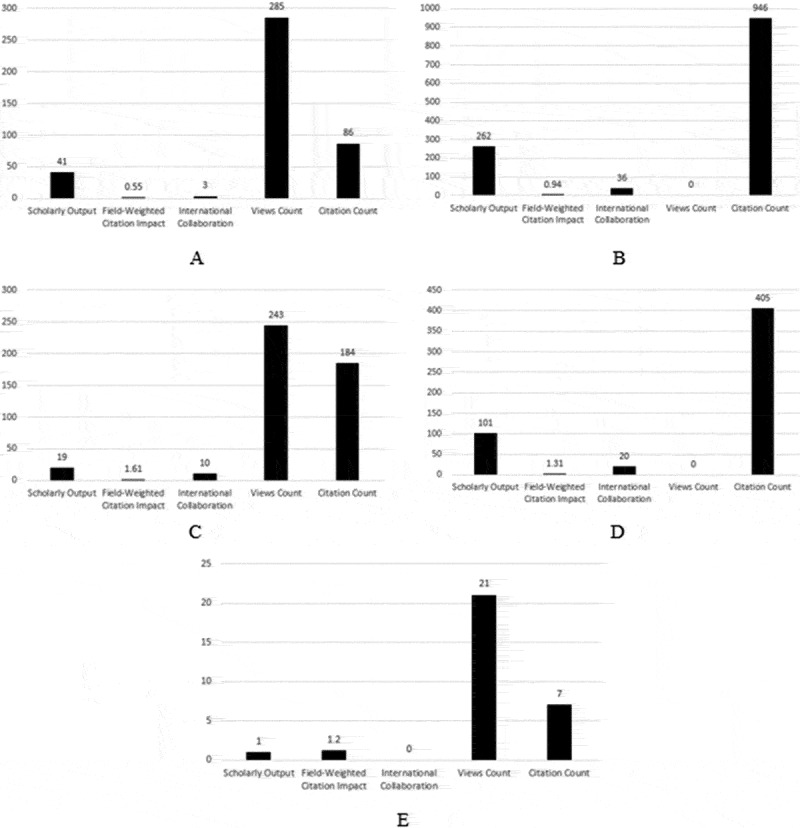
Statistical diagrams represent the scholarly output, field weight citation impact, international collaboration, views count, and citation count of studies on rapid diagnosis of dermatophytosis on the Y-axis and the number of each variant on the X-axis. A. microscopic examination dependent techniques, B. modified rapid culture techniques, C. MALDI-TOF based techniques, D. molecular diagnostic technique, E. lateral-flow based techniques

Dermatophytosis is a disease caused by a group of closely related fungi called dermatophytes. It includes three main genera and more than fifty species. Altogether, there are seven genera *Trichophyton, Microsporum, Epidermophyton, Nannizzia, Paraphaton, Lophophyton, Arthroderma*. All species of the first three genera and some of the *Nannizzia* species are obligate human pathogens. *Microsporum, Trichophyton*, and *Epidermphyton* are these main genera [1]. Of these, the genera *Trichophyton* and *Microsporum* contain zoophilic species. *Nannizzia* is geophilic but can infect animals and humans. The incidence of tinea in humans or ringworm in animals is relatively high, with reinfection and reoccurrence possibilities [2]. It is a highly contagious disease by which 20–25% of all worldwide population infected with it at any time around the year [3] with the highest prevalence of (19.7%) in developing countries [4,5]. Dermatophytosis is conventionally diagnosed using direct microscopic examination, gold standard culturing, and other identification techniques as microcultures and biochemical tests. This is a long, time-consuming process, requiring experts and specific diagnostic protocol [6]. The results were significantly affected by both objective and subjective factors: the amount of the sample, the pre-sampling applied treatments, and the examiner’s experience. Developing a more rapid method or protocol to facilitate the diagnosis of dermatophytosis was the aim of many studies in the past few decades. All developed techniques shared rapidity and showed different levels of specificity and sensitivity. Through the following statistical representation Figure (1), the recent advances in dermatophytosis rapid diagnosis are categorized into five major sections, microscopic examination dependent techniques, modified rapid culture techniques, matrix-assisted laser desorption/ionization-time of flight (MALDI-TOF) based technique, molecular diagnostic techniques, and lateral flow-based techniques. The data is obtained from Scopus databases and analysed using a free author’s analytical services available through Scopus official website “Scival analytics” combined with “Microsoft Excel” for data presentation. The represented data shows the high interest of scientific communities in the recent techniques developed to achieve a rapid diagnosis of dermatophytosis.

## 2. Microscopic examination dependent techniques

### 2.1. Bright-field light microscope

Microscopic examination in dermatophytes infection diagnosis has been used through different diagnostic techniques. Either direct microscopic examination of skin scrapings, hair, scales, and nails using a clarifying agent examined under 40x power of bright field microscope, or histological [7] histopathological [8] and even immunohistochemical examination [9] of biopsied tissue of a lesion from infected cases. Such techniques aim to demonstrate the different hair parasitism arrangements of arthrospores [10]. Several studies and experiments have been tried on a different clarifying agent, during wet slide preparation, to achieve a better cleared microscopic view and less time during the slide preparation and examination. Potassium hydroxide (KOH), sodium hydroxide (NaOH), calcofluor white [11], Chicago sky blue [12], lactophenol cotton blue (LPCB), and a mixture of dimethyl sulphoxide (DMSO) and glycerine with NaOH or KOH.

### 2.2. Phase-contrast light microscope

A better bright-field microscopic view is achieved by contrast and clearance adjustment. That is obtained by lowering the light intensity to achieve maximum contrast and maximum cleared view. So, the phase-contrast microscope will be better because structures are more clearly delineated without loss of light. A 40x phase contrast microscope objective lens has been used to demonstrate the characteristic dermatophytes fungal elements in skin scrapings and nail samples [13].

### 2.3. Fluorescence microscope

Incorporation of fluorescence related techniques in the rapid diagnosis of dermatophytosis has been based on the fact that dermatophytes infected tissue with certain species produce fluorescence when either stained with hematoxylin/eosin (H and E) stain [8,14] or exposed to UV light. On the same bases, Wood’s lamp usage during clinical examination of the cutaneous lesion was developed [15]. The main obstacle facing the natural fluorescence-based dermatophytes technique is this nature’s limitation to a few species leads to overall independence on this technique in dermatophytes diagnosis. On the other hand, using a fluorescent dye as calcofluor white showed significant advances in conventional KOH wet mount [12].

Microscopic examination as a classical step in diagnosing dermatophytosis showed various microscope types and different examination techniques. The most uncomplicated and most rapid technique was using a bright-field microscope in association with one of the clarifying agents. By the time, it showed the lowest specificity, sensitivity, and positive predictive value in comparison to other methods [10].

## 3. Modified rapid culturing techniques

### 3.1. Dermatophytes test medium (DTM)

Other than the conventional isolation of dermatophytes on mycological specific media, modified culturing media can provide a relatively rapid (2 weeks) presumptive identification of dermatophytes if compared with the conventional culturing [16]. Dermatophytes test medium (DTM) is one of those firstly developed media for rapid presumptive identification of dermatophytes [17]. Unfortunately, some non-dermatophytes mould (NDM), either pathogenic or saprophytic, can survive the type and concentration of the used antifungal agents. NDM can also produce alkaline products that change the colour of the media from straw yellow to red. That formed red colour equals or even more intense than the dermatophytes group itself. Moreover, some of NDM may share the dermatophytes group the same shape, site of the lesion, and sample type.

### 3.2. Dermatophytes identification medium (DIM)

A relatively rapid medium for dermatophytes presumptive identification has been developed as DTM modification avoiding the DTM drawback as the non-specific and false-positive NDM reactions [17]. Modifications to DTM were 37°C incubation, and the increased concentration of cycloheximide [18]. This modified DTM is dermatophytes identification medium (DIM) [18]. DIM has been used through certainly developed identification protocol, which showed a great advantage on the DTM in the meaning of sensitivity and specificity. Unfortunately, DIM has high false negatives and false positives, limiting its usefulness and usage [19].

### 3.3. Multi-chromogenic media-dependent protocol

A 2 days protocol is designed for rapid, simple differentiation of two closely similar dermatophytes species [20]. The developed protocol was designed as follows; a 2–20 days old primary isolates were sub-cultured on four different commercially available chromogenic media at different temperatures 4, 20, 25, 30°C with maximum temperature not reach 37°C as temperatures above 37°C would be unsuitable for the growth of most of the dermatophytes group. The reading strategy for those inoculated media was regular inspection from 2 hours to 7 days post incubation. This study [20] showed that Candiselect™ is a promising candidate for achieving rapid and accurate differentiation between the two dermatophyte species studied (within a few hours). Despite the promising results, this technique is only able to distinguish between the two dermatophytes mentioned above. If a different dermatophyte is present, it needs to be further diagnosed, which may take even more time than the traditional culture.

### 3.4. Screening culture-slide method

A thin layer of DTM coats a transparent plastic slide. It principally resembles a rapid microculture technique in which samples are collected and applied on the thin layered media using transparent adhesive tape. The dermatophytes identification depends on micromorphological changes during daily culture-slide microscopic examination in association with some macromorphological colour characteristics [21].

All culturing-based techniques for rapid identification of dermatophytes, either developed for a wide range of dermatophytes identification or only a limited number of species, require a long time that acts as an actual obstacle against a real application of those mentioned studies in the routine diagnosis of dermatophytosis.

## 4. Matrix-assisted laser desorption/ionization-time of flight mass spectrometry (MALDI-TOF) based techniques

This spectrum generation and assessment technique is considered an evolutionary step in the laboratory diagnosis as a highly reliable, accurate, easy to handle, and easy to incorporate in the regular laboratory workflow. At the same time, it overcomes the drawbacks of molecular techniques as high cost, experience needed during the application, and analysis of outcome results or drawbacks of conventional technique as long-time consumption and low specificity [22,23]. MALDI-TOF MS was firstly applied on a whole-cell scale, a fresh culture of suspected microbial colonies. By the time, it became able to deal directly with another type of culture, than solid agar plates as blood cultures and other applications have been incorporated in its system to improve its ability to perform a wide antimicrobial panel [24].

All systems installed for MALDI-TOF identify a wide scale of different bacteria and yeast species level [25,26]. Several trials have been done to apply such a technique in different identification protocols of moulds including dermatophytes [27]. All showed impactive results only when it was associating a laboratory-made database, which is laborious and time-consuming in most cases [28], with a trial to extend the existing MALDI-TOF fungal knowledge database to allow the better robust identification of clinically relevant dermatophytes [29], different studies showed an extensive range of accuracy 13.5% to 100% due to inconsistencies concerning critical steps of the routine pre-analysis preparation laboratory process [29–38]. According to the obtained results, some studies showed that MALDI-TOF is an excellent complementary for conventional culturing in routine dermatophytes diagnosis [29,33,36,39–41].

## 5. Molecular diagnostic techniques

### 5.1. Conventional PCR

Conventional PCR is the most straightforward format of a polymerase chain reaction (PCR) for a highly specific, sensitive, and accurate diagnosis. PCR in dermatophytosis diagnosis able to achieve the diagnostic level of dermatophytes detection or dermatophytes species identification through pan fungal, dermatophytes primer, or species-specific primers respectively [42,43]. Internal transcribed spacer (ITS) and 28s ribosomal DNA are the most used pan dermatophytes primers. They are followed by further outcome amplicon size determination using gel electrophoresis. Despite the sample size, condition, and preparation before applying the PCR reaction, even in mixed cultures, it showed a higher rate of identification than other conventional methods [44–48]. PCR results were affected by the animal species from which the sample was collected as animal behaviour differences affect dermatophytes nucleic acid load on the collected sample, which, by its role, reflected on the results [48]. Although the relatively low cost of conventional PCR compared to other molecular techniques, it requires post-amplification steps and cannot illustrate the situation of infection quantitively [49].

### 5.2. Quantitative/real-time PCR (qPCR)

Quantification of the fungal load of the infected sample can be achieved through the highly accurate, sensitive, and specific quantitative PCR. Real-time PCR depends on a pair of primers and labelled probes targeting pan or species-specific genes. Also, it showed less vulnerable steps to contamination due to less post-amplification handling [42,43]. Although its high cost, it showed a relatively affordable cost in case of high working routine laboratories that examine several samples per cycle [50]. qPCR is a useful diagnostic method in case of treatment effect follow up, differentiation between clinical infection and contamination by establishing a dermatophytes infection threshold. It showed higher sensitivity and specificity characteristics when compared with conventional methods [51–56]. Overall, qPCR showed a significantly higher sensitivity in dermatophytes diagnosis, species identification [53], and differentiation even in the presence of other keratinophilic NDM species in the same site of infection. Generally from previous studies, we cannot depend on qPCR as a complete replacement to the gold-standard method as it showed both false positive and false negative results as it is affected by several factors as sample quality, differences in molecular targets, the used amplification methodology, and the DNA extraction protocols [57–59].

### 5.3. Nested PCR

Aiming to increase PCR specificity in dermatophytosis diagnosis, several targets nested PCR has been set up, especially in pan primers usage [60,61]. A primary amplicon resulting from the first pair of primers amplification cycle will be subjected to another cycle with another primer pair. In the second cycle, the resulted primary amplicon will act as a template for the second pair of primers. The overall sensitivity of nested PCR set was very high in several previous studies [49,62,63] incorporate a pan PCR protocol for dermatophytes diagnosis, also in comparison to the results obtained from KOH mount slide [64,65] and conventional culturing especially in case of that cultures which fail to achieve macroscopic growth [66]. However, the incidence of contamination and time consumption in comparison to the one set up PCR act as the main disadvantage in its application [2,61,67].

### 5.4. Multiplex PCR

One of those PCR techniques developed to be used in case of sample limitation with the need to detect multiple causative agents in the same sample using the same PCR reaction using two or more sets of primers. The nucleotide sequences of each forward and reverse primer in each chosen set should be checked for dimerization, which may lead to unspecific amplification. Several multiplex/duplex PCR have been designed [53] and tried multiple times [44,45] and showed a competitive sensitivity and specificity than culturing and KOH wet mounts with the highest accuracy in cases of onychomycoses and *T. rubrum* [51,67].

### 5.5. PCR-ELISA

A hybrid technique based on both PCR and Enzyme-linked immunosorbent assay (ELISA) in which a labelled nucleic acid amplicon is used instead of target analyte protein in microculture ELISA plates [68]. To increase its sensitivity, a specific probes hybridization was performed before ELISA application. Two hybridization formats have been developed. Either amplification of target genes in the presence of digoxigenin and biotin-labelled nucleotide probe to form a biotinylated PCR amplicon [69]. The fixed amplicon on the microtiter plate was detected using anti-digoxigenin peroxidase [70]. The other format is based on amplification in the presence of a fluorescently labelled nucleotide probe, which is detected using horseradish peroxidase-conjugated anti fluorescein antibodies [71]. Although time consumption and exhaustive application, it was of low cost per sample compared to qPCR [50], and several designs have been developed through various studies [72–74]. However, it is not considered the most applicable technique in dermatophytes routine diagnosis due to its laborious effort and time consumption compared to other established methods.

### 5.6. PCR-Restriction fragment length polymorphism (PCR-RFLP)

PCR-RFLP is another hybrid technique based on PCR and further restricted fragment length polymorphism step using restriction enzymes [75,76]. To a certain extent, it resembles a low-cost replacement of nested PCR as it was able to detect different dermatophytes species and even different NDM species from a multi-causes’ onychomycoses cases [61,77–81] but could not detect variation within the same species [82,83]. Principally, this assay depends on the amplification of target sequences, which shows species-specific variation; the amplicon was then treated with specific restriction enzymes; finally, amplicon sequencing or gel electrophoresis step was performed to compare the molecular weight of the bands to standard ladder [61,79]. However, it seems a relatively low cost, easy to be performed design and higher sensitivity than conventional culture methods [84]. Due to this technique’s laborious, exhaustive, and time-consuming nature and restriction enzyme requirement, it is excluded from the routine dermatophytes’ diagnosis scheme [85].

Other molecular assays have been developed or under development in the field of rapid, molecular dermatophytosis diagnosis. Mainly based on or hybrid with a PCR step as PCR – high resolution melt assay (PCR-HRM) [86], PCR-pyrosequencing [87], single primer-PCR [88], arbitrary primed-PCR [89], PCR-reverse line blot [90], fingerprinting [91], genomic or oligonucleotide array [92] and proteomic analysis techniques [92,93], isothermal amplification techniques [94] and random primer amplification polymorphic DNA (RAPD) [95]. All showed promising results on the research levels, not for routine clinical use.

## 6. Lateral flow-based techniques

Lateral flow-based techniques have been developed for rapid detection of various infectious and analytical agents during the past few years [96] that meets the affordable, sensitive, specific, user-friendly, rapid and robust, equipment-free, and deliverable to end-users (ASSURED) criteria of the world health organization (WHO) [97]. A lot of lateral flow assays with different accuracy, specificity, and sensitivity were developed. Immunochromatography lateral flow assay (ILFA), nucleic acid lateral flow immunochromatographic assay (NALFIA) [98] and nucleic acid lateral flow assay (NALFA). NALFA can be based either on heterothermal amplification (PCR-NALFA) [99,100] or isothermal amplification (RPA-NALFA) [101]. Prospectively, Clustered Regularly Interspaced Short Palindromic Repeats/*cas*12 lateral flow assay (CRISPR/*cas*12-LFA) [102,103] could be developed for diagnosis of dermatophytes. CRISPR/*cas*12-LFA developed either using the DETECTR system [104,105] or SHERLOCK system [106–110]. Each lateral flow assay can be developed in different formats, typical sandwich, competitive, and multiplex [111]. Few studies that have been done as a preliminary step in applying a lateral flow-based technique considered this assay as a rapid method of dermatophytes identification.

In a single-arm comparative study through which a dermatophyte has been detected within 222 samples, a comparison between the conventional direct microscopic examination, highly specific PCR, and a recently developed monoclonal antibody-based lateral flow was performed to diagnose onychomycosis. The accuracy obtained from the three compared methods was as follows sequentially; 90.5%, 76.6%, and 92.5%, while 45 samples showed the difference in the obtained results accuracy between the three compared methods, were relied on PCR result as a conclusive judgement [112].

Further investigations [113] showed that a dermatophyte lateral flow kit is the prober candidate as a rapid diagnostic technique, replacing the highly advanced complex to deal with molecular techniques and the conventional long-time consuming techniques.

Till the current date, there are no polyclonal antibodies, and all trials were for monoclonal antibodies-based lateral flow kit for rapid diagnosis of dermatophytosis [60,112–116]. Such a polyclonal antibodies – based lateral flow kit will achieve the complicated formula characterized by rapidity, field applicability, and low cost of production as the only problem facing the monoclonal based lateral flow is the high cost of development and production.

## 7. Conclusion

Several successfully developed diagnostic techniques for dermatophytosis are now available to substitute the conventional methods. A lot of those techniques are more reliable and more impactive on the diagnosis of dermatophytosis. Some showed high-cost problems or requirements of experts; others showed quite sensitivity, specificity, or rapidity, others need a lengthy pre-examination preparation. Nowadays, the lateral flow-based techniques are considered a promising competitor to both conventional laboratory-based diagnostic methods and advanced molecular-based diagnostic techniques. Enhancing the lateral flows’ diagnostic accuracy, sensitivity, and specificity is the recent challenge and prospect for dermatophytosis diagnosis.
